# Inflammasome Signaling Regulates the Microbial–Neuroimmune Axis and Visceral Pain in Mice

**DOI:** 10.3390/ijms22158336

**Published:** 2021-08-03

**Authors:** Mònica Aguilera, Valerio Rossini, Ana Hickey, Donjete Simnica, Fiona Grady, Valeria D. Felice, Amy Moloney, Lauren Pawley, Aine Fanning, Lorraine McCarthy, Siobhan M. O’Mahony, John F. Cryan, Ken Nally, Fergus Shanahan, Silvia Melgar

**Affiliations:** 1 APC Microbiome Ireland, University College Cork, T12 YT20 Cork, Ireland; monica.aguilerap@gmail.com (M.A.); valerio.rossini@ucc.ie (V.R.); ana.hickey@umail.ucc.ie (A.H.); donjete@hotmail.com (D.S.); fiona.m.grady@gmail.com (F.G.); valeria.felice@hh.global (V.D.F.); amy.keelan@outlook.ie (A.M.); laurenpawley@gmail.com (L.P.); a.fanning@ucc.ie (A.F.); mccarthylorr@gmail.com (L.M.); SOMahony@ucc.ie (S.M.O.); J.Cryan@ucc.ie (J.F.C.); k.nally@ucc.ie (K.N.); f.shanahan@ucc.ie (F.S.); 2 School of Biochemistry and Cell Biology, University College Cork, T12 YT20 Cork, Ireland; 3 Department of Anatomy and Neuroscience, University College Cork, T12 YT20 Cork, Ireland

**Keywords:** gut–brain axis, gut commensal microbiota, immune system, inflammasome

## Abstract

Interactions between the intestinal microbiota, immune system and nervous system are essential for homeostasis in the gut. Inflammasomes contribute to innate immunity and brain–gut interactions, but their role in microbiota–neuro–immune interactions is not clear. Therefore, we investigated the effect of the inflammasome on visceral pain and local and systemic neuroimmune responses after antibiotic-induced changes to the microbiota. Wild-type (WT) and caspase-1/11 deficient (Casp1 KO) mice were orally treated for 2 weeks with an antibiotic cocktail (Abx, Bacitracin A and Neomycin), followed by quantification of representative fecal commensals (by qPCR), cecal short chain fatty acids (by HPLC), pathways implicated in the gut–neuro-immune axis (by RT-qPCR, immunofluorescence staining, and flow cytometry) in addition to capsaicin-induced visceral pain responses. Abx-treatment in WT-mice resulted in an increase in colonic macrophages, central neuro-immune interactions, colonic inflammasome and nociceptive receptor gene expression and a reduction in capsaicin-induced visceral pain. In contrast, these responses were attenuated in Abx-treated Casp1 KO mice. Collectively, the data indicate an important role for the inflammasome pathway in functional and inflammatory gastrointestinal conditions where pain and alterations in microbiota composition are prominent.

## 1. Introduction

The resident microbiota of the gut plays a critical role in regulating the physiology, metabolism, immune function, and nutrition of the host. Changes in microbiota composition are associated with functional and inflammatory gastrointestinal disorders, including irritable bowel syndrome (IBS) and inflammatory bowel disease (IBD) [1,2]. These are multifactorial disorders with alterations in immune and nociceptive responses to several factors, including diet and antibiotics [3,4,5]. Inflammasomes are cytosolic multiprotein complexes of the innate immune system composed of a sensor protein, an adaptor protein, and an inflammatory caspase. They are activated by pathogen-associated molecular pattern molecules (PAMPs) and damage-associated molecular patterns (DAMPs), leading to caspase-1 activation (and caspase 11 in mice), inducing the secretion of the mature forms of the pro-inflammatory cytokines Interleukin (IL)-1β and IL-18 [3,6]. They are expressed in both innate and adaptive immune cells and in non-immune cells, including epithelial cells (enterocytes), microglia, astrocytes and neurons [3,6,7,8]. Recent research shows a bidirectional interplay between inflammasome multiprotein complexes—such as the NLRP3-inflammasome—and the gut microbiota in maintaining intestinal homeostasis, which is altered in intestinal inflammatory and functional conditions (IBD and IBS, respectively) [9,10,11,12,13]. The enteric nervous system (ENS) is a network of neurons and glia essential for gastrointestinal function. Glial dysfunction can contribute to neurological disorders, but its role in digestive disorders remains unclear. Enteric glia cells (EGCs) are located throughout the gastrointestinal tract and closely appose neurons, immune cells, blood vessels and the intestinal epithelium. Mucosal glia, which express components of the NLRP3 inflammasome, encircle crypts thereby forming an intercellular signaling network with epithelial cells [10,14]. Indeed, recent studies have implicated the inflammasome in regulating the gut–brain axis in conditions, such as anxiety, depression and migraine, disorders associated with the activation of central nociceptive responses and pain [15,16,17,18,19]. Moreover, alterations to microbiota composition (by antibiotic treatment or the administration of probiotics) can change the expression of innate pathways such as toll-like receptors (TLRs) and genes underpinning nociceptive and pain responses, including the endocannabinoid, opiate, nerve growth factor and vanilloid pathways [20,21,22,23,24,25]. Collectively, the evidence implicates the inflammasome in local and systemic microbial–neuro-immune responses, including pain activation.

In this work, we investigated the contribution of the inflammasome to the microbiota–neuro–immune axis by using a 2-week antibiotic treatment to perturb the microbiota of wild-type (WT) and Casp1 KO mice followed by an analysis of visceral pain and local and systemic neuro-immune responses. Our data show that Abx-induced microbial alterations lead to increased neuro–immune activation in WT mice, which was attenuated in Casp1 KO mice, implicating the inflammasome as an important regulator of the microbial–neuro–immune axis.

## 2. Results

### 2.1. Antibiotic Treatment Leads to Significant Alterations in Proteobacteria, Actinobacteria and Firmicutes Abundance and in Acetate Levels in Casp1 KO Mice

**Figure 1:**
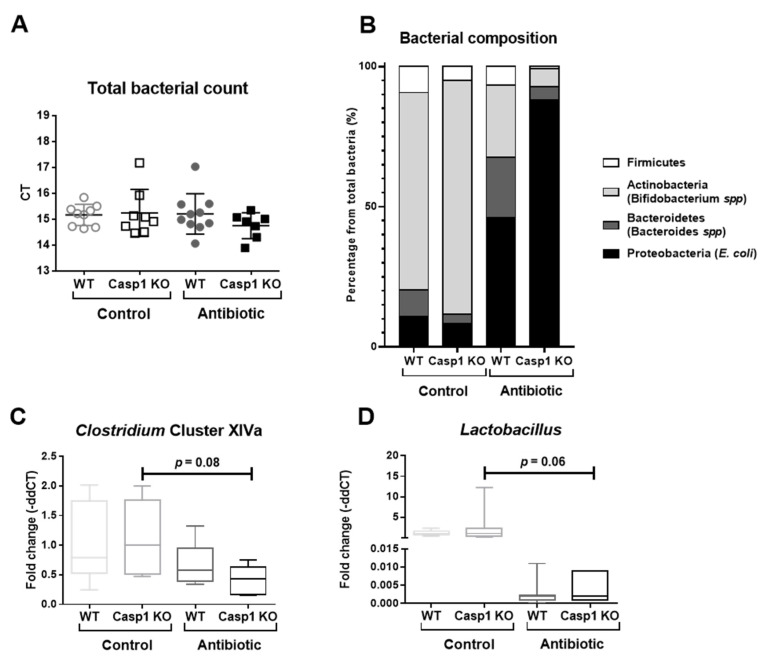
Characterization and quantification of total bacteria, the phylum Firmicutes, Actinobacteria, Bacteroidetes and Proteobacteria, and the genera *Clostridium* XIVa and *Lactobacillus* by qPCR in control and antibiotic-treated WT and Casp1 KO mice. (**A**) Representative cycle threshold (CT) values for total bacterial qPCR detection. (**B**) Percentage (%) of the composition including Firmicutes, Actinobacteria (*Bifidobacterium* spp), Bacteroidetes (*Bacteroides* spp) and Proteobacteria (*E. coli*), representing the commensal microbiota from the total bacterial abundance and (**C**) abundance of *Clostridium* cluster XIVa and (**D**) *Lactobacillus* Order of the Firmicutes phylum. (**A**) Data are presented as mean (SD), *n* = 7–10/group. (**B**) Data are presented as percentage of each phylum from the fold change of the total bacterial abundance. (**C**,**D**) Data are median (interquartile range) (SD).

To validate the alterations provoked by the Abx-cocktail treatment, clinical and macroscopic markers, the expression of representative commensal bacteria in mice [26], including the phylum Actinobacteria (*Bifidobacterium* spp), phylum Bacteroidetes (*Bacteroides* spp), the phylum Firmicutes (*Firmicutes*, *Clostridium* cluster XIVa and *Lactobacilli*) and the phylum Proteobacteria (*E. coli*) were assessed [20,21]. As previously reported in C57BL/6 mice [20,21], Abx treatment led to an increase in the relative weight of the cecum in both WT and Casp1 KO groups (Supplementary Figure S1D). The Abx-treated Casp1 KO group lost 1.2% of their initial body weight (*p* < 0.05), compared to its counterpart Casp1 KO control, while no change in body weight was observed in Abx-treated WT mice (Supplementary Figure S1A). No major impact on the animals’ health status (i.e., hunch posture, fecal consistency or fur texture) were observed in either of the groups (data not shown), and macroscopic markers (colon length and weight) were similar in WT and Casp1 KO control and Abx-treated groups (Supplementary Figure S1B,C). Using primers directed to total 16S, we noticed a similar abundance of total bacteria in WT and Casp1 KO control and Abx-treated groups (Figure 1A). A higher abundance of *Bifidobacterium* spp was seen in Casp1 KO control group compared to the WT control group (*p* = 0.07), with non-significant alterations in the other bacteria phyla and genera analyzed (Figure 1B,C,D; Supplementary Table S1). Consistent with previous reports [20,21], the Abx-treatment in the WT group significantly increased the percentage of *Bacteroides* spp (*p* < 0.05) compared to the WT control group (Figure 1B–D; Supplementary Table S1). In contrast, the Abx treatment in Casp1 KO mice resulted in a reduction in *Bifidobacterium* spp (*p* < 0.05), *Firmicutes* (*p* < 0.0001), *Lactobacilli* (*p* = 0.06) and *Clostridium* XIVa cluster (*p* = 0.08) and a significant increase in *E. coli* (*p* < 0.01) when compared to the Casp1 KO control group (Figure 1B–D; Supplementary Table S1). A significant reduction in cecal acetate was seen in both Abx-WT (*p* < 0.05) and Abx-Casp1 KO (*p* < 0.0001) treated groups compared to their respective control groups (Table 1). Antibiotic treatment non-significantly reduced propionate in WT and Casp1 KO groups, while butyrate levels were increased in Casp1 KO-Abx compared to the WT-Abx group (Table 1). Overall, the data show that antibiotic treatment significantly perturbed the abundance of fecal Proteobacteria, Actinobacteria and *Firmicutes* and reduced cecal acetate levels in Casp1 KO mice.

### 2.2. Antibiotics Alter Intestinal Immune Markers in Casp1 KO Mice

**Figure 2:**
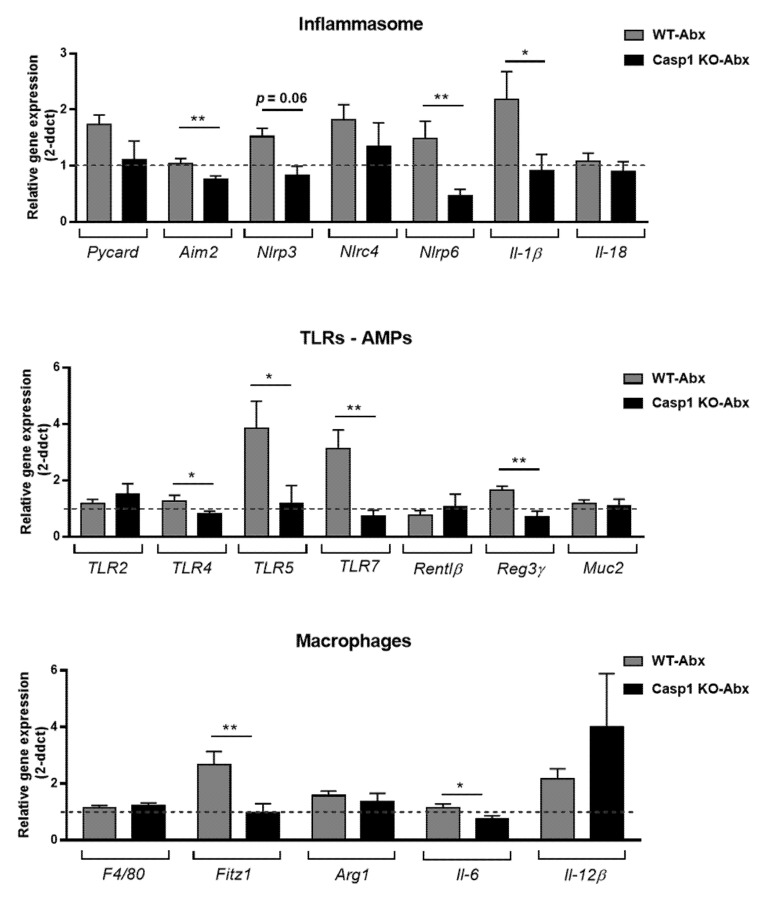
Representative RT-qPCR expression of genes associated with (**A**) inflammasome (*Pycard, Aim2, Nlrp3, Nlrc4, Nlrp6, Il-1β* and *Il-18*); (**B**) toll-like receptors (*TLR2, -4, -5* and *-7*); antimicrobial peptides (AMPs-*Rentlβ* and *Reg3γ*), the mucus layer component *Muc2* and (**C**) macrophage M1/M2 signature (*F4/80, Fitz1, Arg1, Il-6* and *Il-12β*), the colon of WT and Casp1 KO antibiotic-treated groups. Data are mean (SEM). *n*= 7–9/group. *: *p* < 0.05, **: *p* <0.001. Dashed line indicates a background value of 1 of WT and Casp1 KO-control groups. Abx: Antibiotic.

In line with previous reports in C57BL/6 mice treated with the 2-week Abx-cocktail [20,21], alterations in *TLR4,-5,-7* gene expression as well as inflammasome associated genes were evident in the WT-Abx group (Figure 2A,B). However, Abx treatment to the Casp1 KO group reduced the expression of most host-microbe associated genes from the inflammasome, TLR and AMP pathways, with a reduction in *Aim2* (*p* < 0.001)*, Nlrp3* (*p* = 0.06), *Nlrp6* (*p* < 0.001), *Il-1β* (*p* < 0.05) (Figure 2A), *TLR4* (*p* < 0.05)*, TLR5* (*p* < 0.05)*, TLR7* (*p* < 0.001) and *Reg3γ* (*p* < 0.001) (Figure 2B) compared to the WT-Abx group. With regard to macrophage-associated gene expression signature, a significant reduction in *Fitz1* (*p* < 0.001) and *Il-6* (*p* < 0.05) (M2 and M1 marker, respectively) was seen in the Abx-treated Casp1 KO group compared to the WT group counterpart (Figure 2C). In addition, significant reductions in the expression of mKC (a neutrophil attracting chemokine, *p* < 0.01) and the T cell markers (CD3 (*p* < 0.001), CD4 (*p* < 0.01) and CD8 (*p* < 0.05)) was detected in Abx-treated Casp1 KO group compared to the WT-Abx treated group (Supplementary Figure S3).

**Figure 3:**
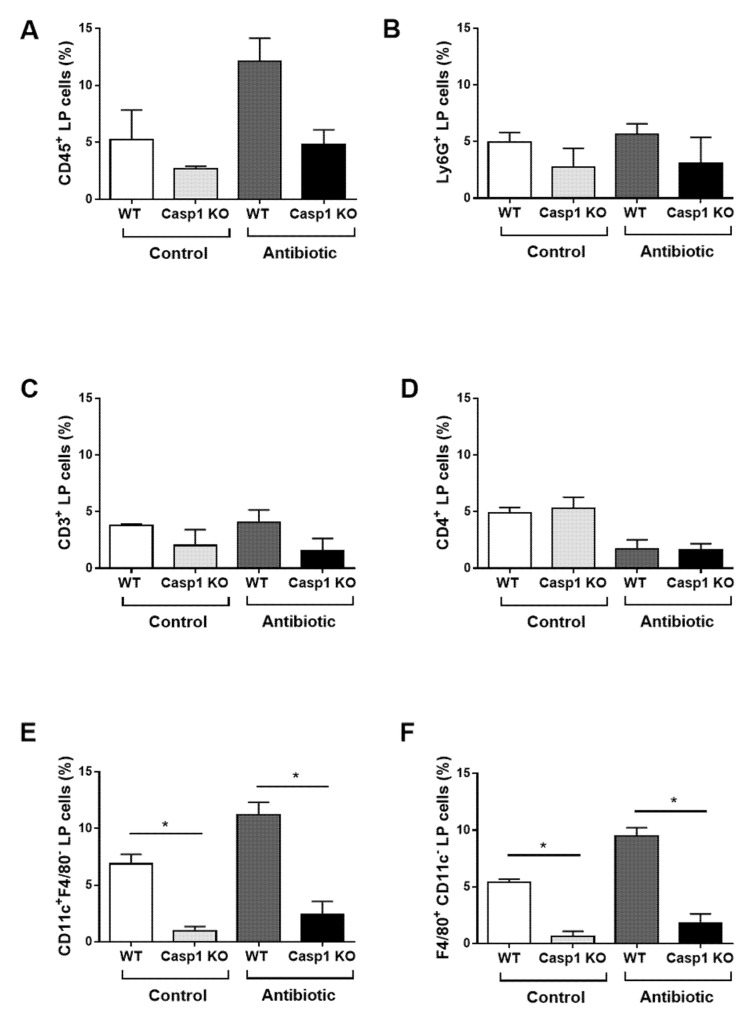
Colonic lamina propria (LP) cells isolated from control and antibiotic-treated WT and Casp1 KO mice and stained with (**A**) CD45 (Leukocytes); (**B**) Ly6G (neutrophils); (**C**) CD3 (CD3^+^ T cells); (**D**) CD4 (CD4^+^ T cells); (**E**) CD11c (dendritic cells); and (**F**) F4/80 (macrophages). Bars represent the percentage of the indicated cell population. Representative figure of two individual experiments. Data are mean (SEM). *n* = 4–5/group. *: *p* < 0.05.

Immunophenotyping of colonic lamina propria (LP) cells isolated from control and Abx-treated WT and Casp1 KO mice showed that the number CD45^+^ (leukocytes), neutrophils (Ly6G^+^), T cells (CD3^+^), dendritic cells (DCs, CD11c^+^/F4/80^−^) and macrophages (CD11c^−^/F4/80^+^), were reduced in Casp1 KO compared to the WT control group (Figure 3A–C,E,F). Abx-treatment was associated with a similar number of colonic LP CD4^+^ T cells and neutrophils and an increase in CD45^+^ cells, DCs (CD11c^+^/F4/80^−^) and macrophages (CD11c^−^/F4/80^+^) in the WT group but not in Abx-treated Casp1 KO group (Figure 3A,B,D–F). Overall, the data indicate an attenuation in intestinal immune responses in Casp1 KO mice with antibiotic-induced microbial changes.

### 2.3. Antibiotic-Induced Changes Are Linked with Reduced Gut–Brain Neuro and Pain Responses in Casp1 KO Mice

**Figure 4:**
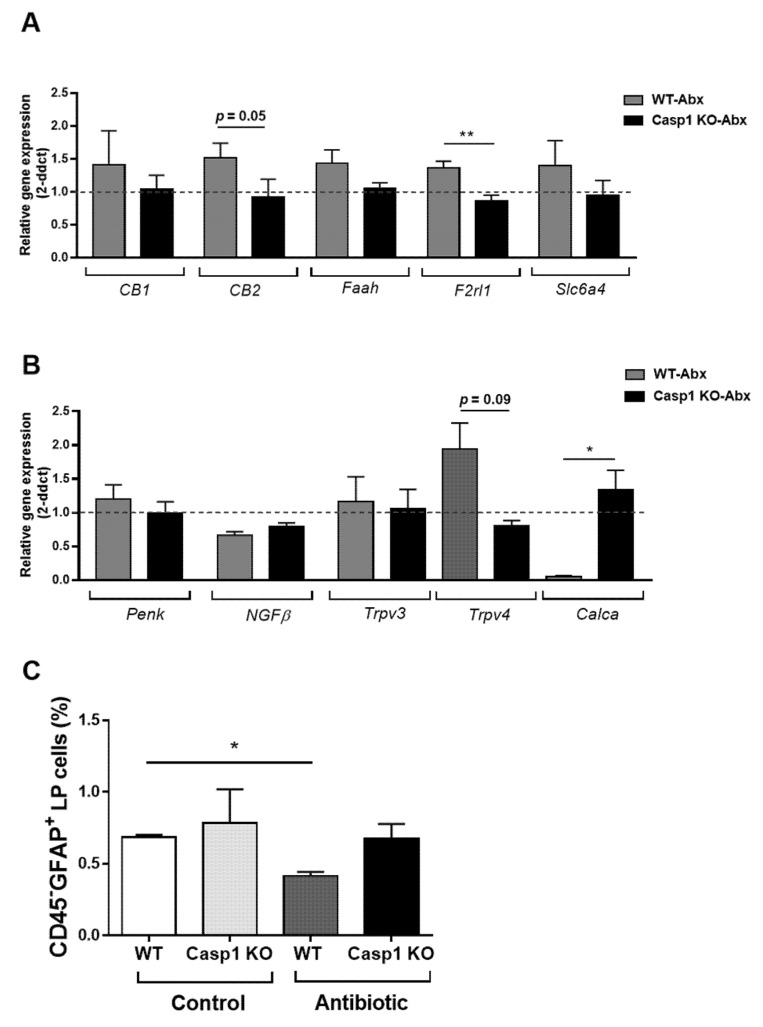
Representative RT-qPCR expression of colon nociceptive markers; (**A**) the endocannabinoid system (*CB1, CB2 and Faah*), the protease-activated receptor 2 (PAR2, *Fr2l1*) and the serotonin transporter (*Scl6a4*); (**B**) the opioid peptide pro-enkephalin (*Penk*), the neurothrophin (NGFβ), the vanilloid system (transient receptor potential, *Trpv3 and Trpv4*) and calcitonin-related polypeptide alpha (*Calca*). Data are mean (SEM). *n* = 7–9/group. *: *p* <0.05, **: *p* < 0.001. Abx: antibiotic. Dashed line indicates background value of 1 of WT and Casp1 KO-control groups. (**C**) Colonic lamina propria (LP) cells isolated from control and antibiotic-treated WT and Casp1 KO mice and stained with CD45 and GFAP. Bars represent the percentage of the indicated cell population. Representative figure of two individual experiments. Data are mean (SD). *n* = 4/group. *: *p* < 0.05.

The assessment of RNA expression of selected genes representative of known nociceptive pathways in the gut, including endocannabinoid, the protease-activated receptor 2 (PAR2, *Fr2l1*), serotonin and vanilloid families, revealed similar expression levels in the Casp1 KO control compared to the WT control group (Supplementary Figure S4). Genes belonging to the endocannabinoid system (*CB2, p* = 0.05), the protease-activated receptor 2 (*Fr2l1*) (*p* < 0.001) (Figure 4A) and the vanilloid system (transient receptor potential *Trpv4*, *p* = 0.09) (Figure 4B), all of which induced by the Abx-treatment in WT mice, were reduced in expression in the Abx-treated Casp1 KO mice. Similar non-significant gene expression reductions were seen for *CB1, Faah*, and the serotonin transporter (*Scl6a4*) in Abx-treated Casp1 KO mice (Figure 4A,B). In contrast, the expression of calcitonin-related polypeptide alpha (*Calca*) was significantly increased (*p* < 0.05) in Abx-treated Casp1 KO mice compared to the WT-Abx treated group (Figure 4B). In addition, a significant reduction in the number of GFAP^+^ enteric glial cells was found in the Abx-treated WT-mice (*p* < 0.05) but not in the Abx-treated Casp1 KO group (Figure 4C).

**Figure 5:**
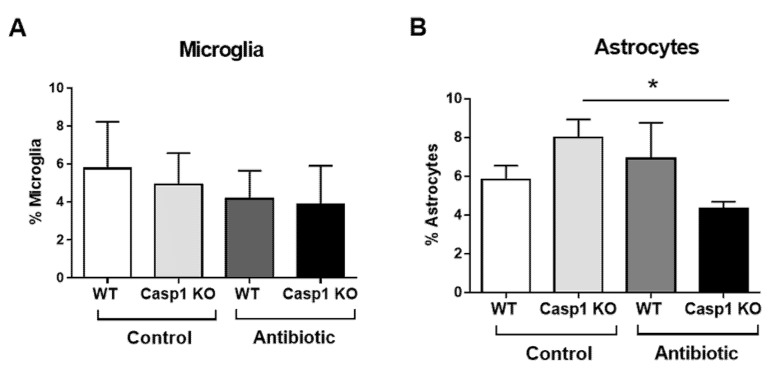
Representative percentage (%) of (**A**) microglia and (**B**) astrocytes cell average in the anterior cingulate cortex (ACC) of WT and Casp1 KO control and antibiotic-treated groups. Data are mean (SD). *n* = 3/group. *: *p* < 0.05.

When the presence of astrocytes (GFAP) and microglia (Iba1) cells was analyzed in the brain, no major changes were observed for microglial density in the anterior cingulate cortex (ACC) regardless of genotype or treatment. When assessing the astrocyte density in ACC, a significant reduction was observed in the Casp1 KO Abx-treated group compared to the WT group (*p* < 0.05; Figure 5).

**Figure 6:**
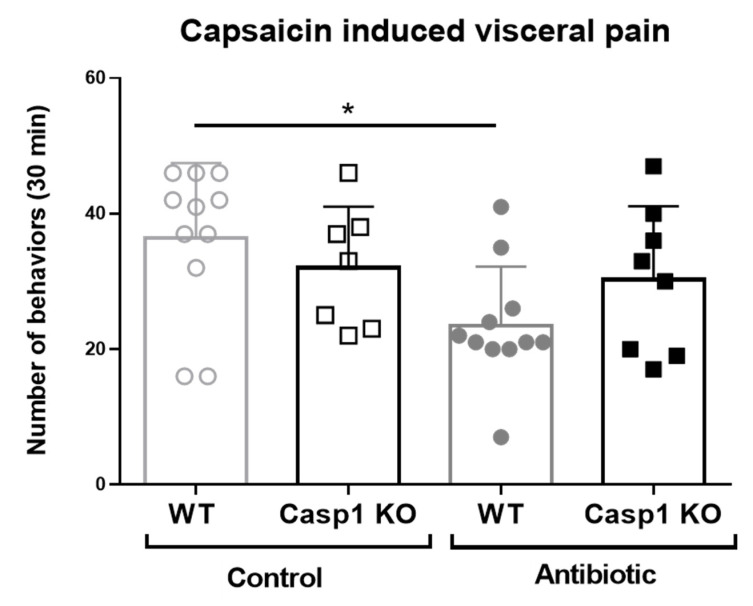
Representative number of visceral pain behaviors during 30 min per each experimental group upon intracolonic administration of capsaicin. Data mean (SD), * *p* < 0.05 compared to the WT control group. *n* = 7–11/group, including in all groups, males (range 2 to 8) and females (range 3 to 5).

Our above findings indicated that the response to pain in Abx-treated Casp1 KO mice is altered, which is why a behavioral study using capsaicin was conducted. Capsaicin installation induced pain-related behaviors in all mice during the 30 min observation period. The predominant behaviors included the licking of the abdomen, stretching of the abdomen and squashing of the lower abdomen against the floor, all considered to reflect maximum levels of painful behavior observed [18]. Capsaicin-induced pain-related behaviors were reduced by 36% in Abx-treated WT mice (23.5 ± 2.6 behaviors/30 min, *n* = 11, 5 males and 6 females) compared to WT vehicle-treated mice (36.5 ± 3.3 behaviors/30 min, *n* = 11; *p* < 0.05; Figure 6). In contrast, Abx- and vehicle-treated Casp1 KO mice had a similar number of behaviors (33.0 ± 3.4 and 30.2 ± 3.8 behaviors/30 min, respectively) to that of WT-vehicle-treated mice (36.5 ± 3.3 behaviors/30 min). Collectively, the data indicate no attenuation in pain-induced behavior in Casp1 KO mice associated with antibiotic-induced microbial perturbation.

## 3. Discussion

The results confirm a regulatory role for the inflammasome in microbiota-neuroimmune interactions and visceral pain responses. Antibiotic-induced microbial changes resulted in neuro-immune responses and visceral pain attenuation in WT but not in Casp1 KO mice.

Antibiotic induced bacterial changes in WT and Casp1 KO mice were consistent with previous publications using the same Abx-cocktail and regimen [20,21,22] and were accompanied by a significant reduction in acetate levels in Abx-treated WT-mice, which is in agreement with their reduction in acetate producing bacteria, e.g., *Bifidobacteria* spp [27]. Antibiotic treatment of WT mice also led to a high reduction in butyrate (around 80%), and approximately 30% higher level of butyrate in Abx-Casp1 KO mice. Although the main butyrate producers Firmicutes are reduced in these mice, the overall increased butyrate may be explained by the large increment in Proteobacteria, especially in the Abx-Casp1 KO group [27,28]. Furthermore, loss of butyrate might be one of the reasons why the Abx-treated WT group but not the Casp1 KO group exhibited less visceral pain behaviors [29].

SCFAs and especially butyrate have been directly linked to the modulation of the enteric nervous system (ENS), including both neurons, enteric glia cells [30] and microglial cells, although the mechanisms are uncertain [27,31,32,33]. Butyrate appears to induce colonic hypersensitivity via peripheral up-regulation of NGF in animals, and enteric glial cells seem to be an important source of NGF. Although no changes were seen in NGF in either of the genotypes or treatment in this study, a reduction of EGC-GFAP^+^ cells was found in Abx-treated WT mice but not in Abx-treated Casp1 KO mice, potentially due to Caspase-1 dependent cell death in the enteric glial cells in WT mice [34]. The Abx-treated Casp1 KO group demonstrated no pain behavior attenuation and less expression of the inflammation-associated nociceptors CB2, PAR2 and TRPV4. These nociceptors have been shown to regulate the inflammasome pathway in a model of brain injury and demyelination, where the deletion or inhibition of TRPV4 reduced the activation of the NLRP3 inflammasome pathway [35,36]. In addition, administration of CB2 agonists or PAR-2 antagonist to mice with inflammatory pain or with knee joint pain led to the reduction of NLRP3 inflammasome activation [37,38]. Previous studies have widely described the sensitization of these nociceptor pathways in gastrointestinal inflammation by immune mediators [39,40,41,42]. Specifically, our data indirectly indicate that the inflammasome pathway can also regulate the expression of these mucosal nociceptors and their subsequent effect on visceral pain.

Enteric glial cells sense pathogenic bacteria and promote an effective host-immune response involving toll like receptors or inflammasomes [17]. Myenteric GFAP-expressing glial cell subpopulations are particularly susceptible to acute inflammation and modulate mucosal inflammation and homeostasis [14,43]. Changes in the visceral pain response due to antibiotic-induced modulation of the microbiota have been described previously, but the immune mechanisms have not been established [20,21]. Interactions between enteric glia and immune cells are thought to contribute to nociceptor sensitization during inflammation [44]. Furthermore, the influx of macrophages and development of peripheral pain present a direct correlation [45]. Macrophages located at the base of the crypt come in close contact with neurons of the submucosal plexus. A crosstalk between the macrophages present in the muscularis externa layer and neurons of the myenteric plexus has been described [46,47]. Our results are consistent with microbiota–neuro–immune crosstalk, with Abx-treated WT mice having fewer pain behaviors and a decrease in GFAP^+^ cells and an increase in colonic lamina propria macrophages and dendritic cells, all of which is ablated in Abx-treated Casp1 KO mice.

The microbiota has been shown to modulate visceral pain via TLRs. Studies have also shown that visceral pain in the gut is associated with an over-activation of neurons in the anterior cingulate cortex [48], which is one of the main brain areas involved in pain processing in rodents. The involvement of the inflammasome pathway in the gut–brain axis was previously described for depressive-like behaviors following chronic restraint stress [15] in neurons expressing the IL-1β receptor and by IL-1β serving as a mediator in nociceptive responses and pain [17,18,19,49]. In the present work, we extended those findings by showing that the inflammasome is a regulator of the gut–brain-microbiota axis, as demonstrated by the reduced number of astrocytes in the ACC area of Abx-treated Casp1 KO mice and by the absence of a decrease in visceral pain responses seen in Abx-treated WT animals. Further studies should examine whether changes in nociception markers in the ACC are also regulated by the inflammasomes and microbiota.

Limitations of this study include the low number of samples in some of the analyses and the restricted characterization of the intestinal microbiota composition upon antibiotic treatment and Casp1 deficiency. Future studies could address a full characterization of the intestinal microbiota by 16S rRNA or metagenomic analysis at the basal state and upon antibiotic treatment in Casp1 KO mice to potentially correlate specific microbial taxa regulated by the inflammasome and involved in neuro-immune responses.

As key sensors detecting different danger signals, such as inflammatory stimuli and bacteria, the inflammasomes, and particularly the NLRP3 inflammasome, have been associated with a wide range of conditions, including IBD, Parkinson, type II diabetes, etc. [50,51] Preclinical models of intestinal inflammation, chronic pain and others, treated with inflammasome inhibitors have generated promising findings to pursue the next generation of inflammasome inhibitors into clinical trials [50,51,52]. Our findings add intestinal inflammatory and functional disorders with alterations in the microbiota composition, immune system, and pain to the ever-growing conditions that can be therapeutically targeted by the inflammasomes.

In conclusion, the present study demonstrated that Abx-induced alterations in the commensal microbiota and SCFAs (namely acetate and butyrate) led to changes in gut mucosal innate immune cells (macrophages and dendritic cells) and the enteric nervous system, provoking a local immune and nociceptive response and decreasing visceral pain responses. This microbial–neuro-immune interaction is reduced when the inflammasome pathway is blunted, suggesting an important role for this pathway in conditions where pain and alterations in the microbiota composition feature heavily, including functional and inflammatory gastrointestinal disorders [53].

## 4. Materials and Methods

### 4.1. Experimental Groups

Caspase-1/11 knock out (Casp1 KO) was generously provided by Prof. Wolf-Dietrich Hardt, ETH Zurich, rederived to C57BL/6 background at Trinity College Dublin, and bred for at least 3 generations in the animal facility at University College Cork before entering the studies. Eight to eighteen-week-old female and male Casp1 KO and wild type (WT) mice were included in the studies. All animals were maintained in SPF conditions in an environmentally controlled room (20–22 °C, 12 h light:dark cycle), with food and water *ad libitum*. All procedures were approved by the Ethical Committee of the University College of Cork (Ethic approvals 2012/003 and 2011/023) and the Irish Government (DOH license B100/4108).

Randomized mice were treated with the antibiotic mixture (*n* = 20) or vehicle (*n* = 20) for 2 weeks were utilised for qRT-PCT, flow cytometry, SCFAs analysis and histology. Animals (WT-Control *n* = 11; WT-Abx *n* = 10; Casp1 KO-Control *n* = 7; Casp1 KO-Abx *n* = 8) used to assess visceral pain responses (IC capsaicin) were not included in any other analyses. In all cases, all analysis and tissue sampling were performed 24 h after the last Abx/vehicle (deionized water) administration.

### 4.2. Antibiotic Treatment

Animals received daily a mixture of non-absorbable, broad-spectrum antibiotics following a similar treatment protocol previously published in comparable studies in C57BL/6 mice and demonstrating the induction of significant changes of the gut commensal microbiota [20,21]. Briefly, animals were treated to an Abx-cocktail containing 0.5 g/L Bacitracin A (31626-Vetranal™; Sigma-Aldrich, Dublin, Ireland) and 0.5 g/L Neomycin (N1876-Neomycin trisulfate salt hydrate; Sigma-Aldrich, Dublin, Ireland) in their drinking water for a 2-week period, with the Abx-cocktail replenished twice a week. Amphotericin B (A9528; Sigma-Aldrich, Dublin, Ireland) was gavaged for 3 consecutive days at the beginning and at the end of the treatment to prevent yeast overgrowth (1 mg/Kg). Control (vehicle)-treated animals received sterile filtered deionized water. Water consumption and body weight were assessed daily during the Abx-treatment period.

### 4.3. Sample Collection

Mice were euthanatized by cervical dislocation. Thereafter, a medial laparotomy was performed, and the colon was dissected for RNA (frozen in RNA-later, ThermoFisher, Dublin, Ireland) and flow cytometry analysis (see Section 4.4 and Section 4.8). The colon and cecum were weighed, and colon length was measured. The cecal contents were collected for SCFA analysis and fecal pellets were collected for bacteria quantification and both were frozen immediately in liquid nitrogen. Frozen samples were stored at −80 °C until analysis. Brain samples were collected and frozen immediately in liquid nitrogen, or fixed overnight in 4% paraformaldehyde, cryo-protected with 15% and 30% of sucrose (S5016; Sigma-Aldrich, Dublin, Ireland) and frozen with isopentane (M32631; Sigma-Aldrich, Dublin, Ireland). The slides were then returned to −80 °C until immunohistochemical staining.

### 4.4. Isolation of Colonic Lamina Propria Cells

Single cell suspension from colon tissue was obtained using the lamina propria (LP) dissociation kit, mouse (130-097-410; Miltenyibiotec, Dublin, Ireland) following the manufacturer’s instructions. In brief, colon tissues were removed and opened longitudinally and washed 3 times with ice cold 1 X phosphate-buffered saline (PBS, TMS-012; Sigma-Aldrich, Dublin, Ireland) supplemented with 1% fetal calf serum (FCS, 12133C; Sigma-Aldrich, Dublin, Ireland). A maximum of 3 colons, from mice of the same experimental group, were pooled together, with colons cut into 2 × 2 mm pieces, transferred to a tube containing 20 mL of pre-digestion solution (Hanks’ Balanced Salt solution (HBSS without Ca^2+^ and Mg^2+^, H4641; Sigma-Aldrich, Dublin, Ireland) supplemented with 5% FCS, 5 mM ethylenediaminetetraacetic acid (EDTA, 15575020; ThermoFisher Dublin, Ireland), 10 mM HEPES (H0887; Sigma-Aldrich, Dublin, Ireland), and 1 mM dithiothreitol (DTT, R0861; ThermoFisher, Dublin, Ireland) and incubated for 20 min at 37 °C with slow rotation on a horizontal tube rotator. Colon pieces were washed 3 times with 1 X PBS supplemented with 1% FCS, transferred to a new 20 mL pre-digestion solution, and incubated for another 20 min at 37 °C with slow rotation on a horizontal tube rotator. Colon fragments were placed in a tube containing 10 mL of HBSS supplemented with 5% FCS and incubated for 10 min at 37 °C with slow rotation on a horizontal tube rotator. Intestinal pieces were transferred to gentle magnetic-activated-cell-sorting (MACS) C-tubes (Miltenyibiotec) containing 2.5 mL of digestion solution (HBSS with Ca^2+^ and Mg^2+^ supplemented with 5% FCS) supplemented with enzymes provided with the kit and incubated at 37 °C for 30 min with rotation on a horizontal tube rotator. The gentleMACS C-tubes were transferred into the gentleMACS Octo Dissociator (Miltenyibiotec, Dublin, Ireland), and the program 37C_m_LPDK_1 was selected. After program completion, cells were collected, washed twice with 1 X PBS supplemented with 1% FCS, and centrifuged for 10 min at 300 x g. Isolated colonic LP cells were incubated with gentamicin (50 µg/mL) for 30 min at 37 °C to kill extracellular bacteria and then washed 3 times with 1 X PBS.

### 4.5. Bacterial qPCR

Total DNA was isolated from frozen fecal pellets using QIAampDNA Stool Mini Kit (51504; Qiagen, GmbH, Hilden, Germany), following the manufacturer’s instructions. Subsequently, DNA was quantified using the NanoDrop ND-1000 spectrophotometer (Isogen Life Science, De Meern, The Netherlands), diluted to equal concentrations with sterile deionized water and stored at −20 °C until analysis. Quantitative real-time PCR was performed with an ABI prism 7900HT from Applied Biosystems (Nærum, Denmark). All amplification reactions were carried out in transparent 384-well MicroAmp^®^ Optical reaction plates (4309849; ThermoFisher, Diblin, Ireland) and sealed with MicroAmp^®^ Optical Adhesive Film (4311971; ThermoFisher, Diblin, Ireland) in a total volume of 11 µL containing 5.5 µL 2 × SYBR Green PCR Master Mix (FisherScientific, Dublin, Ireland), 0.4 µL of each primer (10 µM), 2 µL template DNA (2 ng), and 2.7 µL nuclease-free water (4387936; ThermoFisher, Dublin, Ireland). Liquid handling was performed with an epMotion 5075 (Eppendorf, Hørsholm, Denmark). The amplification program was identical for all amplifications and consisted of one cycle at 50 °C for 2 min; one cycle at 95 °C for 10 min; 40 cycles at 95 °C for 15 s and 60 °C for 1 min; and finally, a dissociation curve analysis for assessing amplicon specificity (95 °C for 15 s, 60 °C for 15 s, then increasing to 95 °C at 2% ramp rate).

Quantification of the bacteria qPCR was done following previous publications [54]. The WT control group served as the calibration group. Total bacterial PCR primers were used, as well as the endogenous control gene. See Supplementary Table S2 for specific primers and probes.

### 4.6. Bacterial Metabolites (SCFAs)

Cecal samples were defrosted and diluted at ratio 1:10 (*w/v*) in sterile distilled water (HPLC water) −100 mg in 1 mL volume. The SCFA-containing supernatant was filtered through cellulose acetate membrane with a pore size of 0.2 μm (GyroDisc CA; Orange Scientific, Braine-l’Alleud, Belgium) and stored at −20 °C until HPLC analysis. Quantification of SCFAs in fecal samples was carried out using an external calibration standard curves method [55]. Six calibration standards were prepared at five levels of concentration, i.e., 50, 10, 5, 1 and 0.5 mM. The calibration curves were constructed by plotting the relative peak area versus the molarity of the solution. Fecal SCFA concentrations were expressed as mean μmol per gram wet weight cecum using the following equation: Cecal SCFA (μmol/g) = [organic acid in cecal contents (mmol/mL) X Vd (ml) × 1000]/wet weight cecum (g), where Vd = total volume of dilution. Total SCFA was calculated by using the sum of acetic acid, butyric acid, and propionic acid.

### 4.7. Colon RT-qPCR

RNeasy Mini kit (74004; Qiagen, GmbH, Hilden, Germany) was used for the extraction of total RNA for colon samples. The quantification of RNA was measured by using NanoDrop ND-1000 spectrophotometer. The extracted RNA was treated with turbo-DNAfree (AM1907; ThermoFisher, Dublin, Ireland). Reverse Transcription (RT)-qPCR was then performed using 500 ng of RNA. RT-qPCR was performed in a cDNA LightCycler (LC) 480 (Roche Applied Science, Indianapolis, IN, USA). The endogenous control (housekeeping gene) was beta-actin. The calibrator group was the WT control group. ddCT was used to calculate changes. Primers and probes used for PCR analysis were designed using Universal ProbeLibrary Assay Design Center (https://www.roche-applied-science.com/sis/rtpcr/upl/adc.jsp; accessed on 3 April 2021; Roche Applied Science, Indianapolis, IN, USA) [56].

### 4.8. Flow Cytometry

Isolated colonic LP cells were washed twice in PBS/0.3% *w/v* bovine serum albumin (A7030; ThermoFisher, Dublin, Ireland) supplemented with 0.1%*w/v* sodium azide (S2002; ThermoFisher, Dublin, Ireland). Nonspecific binding of antibody (Abs) to Fc receptors was blocked by preincubation of cells with monoclonal antibody (mAb) 2.4G2 directed against the FcgRIII/II CD16/CD32 (0.5 ng mAb per 10^6^ cells). Cells were washed and incubated with 0.5 ng per 10^6^ cells of the relevant mAbs for 20 min at 4 °C and then washed twice. The following reagents and mAbs were used: PerCP-conjugated mAb binding F4/80 (123126), PerCP-conjugated mAb binding CD3 (100325), allophycocyanin (APC)-conjugated mAb binding CD45 (103112), fluorescein isothiocyanate (FITC)-conjugated mAb binding CD4 (100510), all from Biolegend Inc (Biolegend Europe, Amsterdam, Netherlands; FITC-conjugated mAb binding CD11c (557400), PE-conjugated mAb binding GFAP (561483) all from BD (Wokingham, Berkshire, UK). Multicolor flow cytometry analyses were performed using a FACS Calibur flow cytometer (BD Biosciences, Berkshire RG41 5TS, England). Data were analyzed using FCS Express V5 software.

### 4.9. Brain Histology

Brain sections (10 µm) were cut with a Cryostat (Leica, Ashbourne, Co Meath, Ireland) and immediately transferred onto gelatin-coated slides. The brain sections were blocked in 3% donkey serum blocking solution (G9023; Sigma-Aldrich, Dublin, Ireland) prepared in PBS supplemented with 0.1% Triton-X (X100; Sigma-Aldrich, Dublin, Ireland) for two hours at room temperature, followed by overnight incubation at 4 °C with antibodies against GFAP (1:100, Z0334; Dako-Agilent Technologies Ireland Limited, Little Island, Ireland) and IBA1 (1:500, ab5076; Abcam, Amsterdam, the Netherlands). The following day, the sections were washed in PBS (3 times for 10 min) followed by incubation with secondary antibodies conjugated to donkey anti-rabbit IgG (AlexaFluor 488, Life tech, A2120, Thermo Fisher Scientific, Dublin, Ireland) or donkey anti-goat IgG (AlexaFluor 594, Life tech, A11058, Thermo Fisher Scientific Dublin, Ireland) for 2 h at room temperature. Sections were then washed and counter-stained with bizbenzimide (1:3000, B2261; Sigma-Aldrich, Dublin, Ireland).

### 4.10. Image Analysis

Fluorescent labeling was visualized at magnifications of 10x and 20x using an Olympus BX53 fluorescent microscope, and images were analyzed using Olympus CellSens Entry software. Image J software (Rasband, W.S., ImageJ, U. S. National Institutes of Health, Bethesda, MD, USA, https://imagej.nih.gov/ij/, accessed on 3 April 2021; 1997–2016) was used for quantification of the percentage of microglia and astrocytes in the left and right anterior cingulate cortex (ACC).

### 4.11. Behavioral Responses to Intracolonic (IC) Capsaicin-Evoked Visceral Pain

Spontaneous visceral pain-related behaviors induced by intracolonic capsaicin (12084; Sigma-Aldrich, Dublin, Ireland) were assessed following previously described protocols [20,21] Mice were anesthetized with isoflurane (Isoflo; Esteve, Barcelona, Spain) and capsaicin (0.05 mL/mouse, 0.1% in ethanol:Tween 80:saline; 1:1:8, v:v:v;) or vehicle (ethanol:Tween 80:saline; 1:1:8, v:v:v), which were administered intra-colonically. Petroleum jelly was applied to the perianal area to avoid the stimulation of somatic areas due to any leakage of the capsaicin solution. Upon recovering consciousness, visceral pain-related behaviors (licking of the abdomen, stretching the abdomen, squashing of the lower abdomen against the floor or abdominal retractions) were assessed during a 30 min period (in blocks of 5 min), followed by fecal pellet count assessment. Results are the sum of all behaviors during the 30 min. After in vivo evaluation, the animals were euthanized by cervical dislocation. Two independent researchers (M.A. and V.F.), in a blinded fashion, visually assessed pain behaviors.

### 4.12. Statistical Analysis

Data are presented as mean (SD) unless otherwise stated. One-way ANOVA with a multi-comparison Tukey test was performed. For RT-qPCR, the nonparametric Mann–Whitney test was used. Data were considered statistically significant when *p* < 0.05. Graphpad Prism v9.0.0 was used.

## 5. Conclusions

The current study reports that the inflammasome plays a role in regulating microbial–neuro-immune interactions in the gut, as demonstrated by the attenuated neuro-immune interactions and blunted visceral pain detected in Casp1 KO mice with antibiotic-induced microbial alterations. Our findings support the notion of the inflammasome as a promising therapeutic target in the ever-growing list of disorders including intestinal inflammatory and functional conditions where alterations in the microbiota composition and immune system and pain occurs.

## Figures and Tables

**Table 1:** Cecal levels of total SCFAs, acetate, butyrate and propionate (mM/mg) in WT and Casp1 KO control and antibiotic-treated groups.

	WT Control (mM/mg)	Casp1 KO Control (mM/mg)	WT Antibiotic (mM/mg)	Casp1 KO Antibiotic (mM/mg)
Total SCFAs	109.4 ± 37.7	90.4 ± 30.0	51.2 ± 0.1	83.0 ± 41.2
Acetate	41.6 ± 1.6	41.6 ± 5.8	27.1 ± 1.3 *	20.0 ± 2.5 ***
Butyrate	50.6 ± 34.5	32.0 ± 22.9	10.4 ± 1.4	42.4 ± 30.6
Propionate ^$^	17.3 ± 1.6	16.8 ± 2.1	13.7 ± 0.3	13.3 ± 1.4

Data are mean (SD). *: *p* < 0.05 vs. WT Control group. ***: *p* < 0.0001 vs. Casp1 KO Control group. ^$^: *p* < 0.05 general ANOVA difference. *n* = 2 WT Control and WT-Antibiotic groups; *n* = 3 Casp1 KO Control group; *n* = 5 Casp1 KO Antibiotic group.

## Data Availability

Not applicable.

## Supplementary Materials

Supplementary materials can be found at https://www.mdpi.com/1422-0067/22/15/8336/s1.

## Abbreviations

Abx	Antibiotics
ACC	Anterior cingulate cortex
DC	Dendritic Cell
EGCs	Enteric glial cells
ENS	Enteric Nervous System
GFAP	Glial Fibrillary acidic protein
HPLC	high performance liquid chromatography
IBD	Inflammatory Bowel Disease
IBS	Irritable Bowel Syndrome
IL	Interleukin
LP	Lamina propria
KO	Knock out
qPCR	Quantitative Polymerase chain reaction
TLR	Toll Like Receptor
WT	Wild type
