# Supplementary material for: Inflammasome Signaling Regulates the Microbial–Neuroimmune Axis and Visceral Pain in Mice
Source: Int J Mol Sci. 2021 Aug 3;22(15):8336. doi: 10.3390/ijms22158336 (PMC8371481; doi:10.3390/ijms22158336)
Supplement: Supplementary file 1 [file ijms-22-08336-s001.zip › ijms-1279433-supplementary.pdf]

Aguilera et al. "Inflammasome Signaling Regulates the Microbial-neuro-immune Axis and Visceral Pain in Mice "

Supplementary Table S1. Percentage of the composition of the microbiota from the fold change from total bacterial counts

	WT Control	Casp1 KO Control	WT Antibiotic	Casp1 KO Antibiotic
Actinobacteria (*Bifidobacterium* spp)	70.23	83.37	25.80	6.54 *
Bacteroidetes (*Bacteroides* spp)	9.59	3.25	21.48 *	4.72
Firmicutes	9.34	5.05	6.59	0.72 ****
Proteobacteria (*E. coli*)	10.79	8.33	46.13	88.01 **

*: $p < 0.05$ WT Antibiotic vs WT Control; Casp1 KO Antibiotic vs Casp1 KO Control group. **: $p < 0.01$ vs Casp1 KO Control group. ****: $p <0 .0001$ vs Casp1 KO Control group.

Supplementary Table S2. Primer and probe sequences used for bacteria qPCR

Gene	Probe	Forward Sequence (5´→ 3´)	Reverse Sequence (5´→ 3´)
Total Bacteria (16s)	cttgtacacaccgcccgtc (FAM)	cggtgaatacgttcccgg	tacggctaccttgttacgactt
Firmicutes	SYBR	ggagyatgtggtttaattcgaagca	agctgacgacaaccatgcac
Bifidobacteria **spp**	SYBR	gcgtgcttaacacatgcaagtc	cacccgtttccaggagctatt
Lactobacilli **spp**	SYBR	tggatgccttggcactagga	aaatctccggatcaaagcttacttat
Clostridium XIVa	SYBR	aaatgacggtacctgactaa	ctttgagtttcattcttgcgaa
Bacteroidetes	SYBR	ggatcatgtggtttaattcgatgat	agctgacgacaaccatgcag
E. coli Uid405	9 (UPL)	ctgatagcgcgtgacaaaaa	cggttcgttggcaatactc

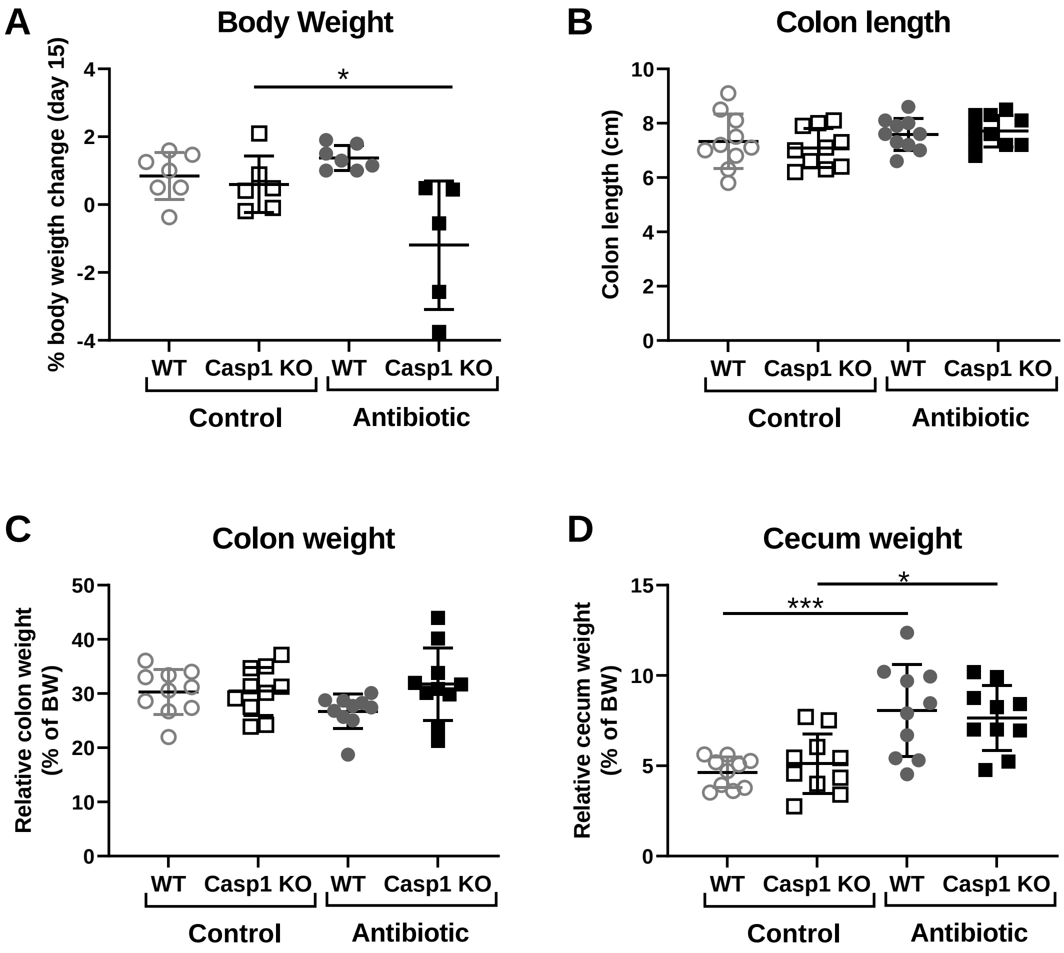

Supplementary Figure S1. Representative graph of (**A**) percentage (%) of body weight change, (**B**) length of the colon, (**C**) percentage (%) of colon weight change and (**D**) percentage of cecum change after antibiotic or vehicle (PBS) treatments in wild type (WT) and Casp1 KO mice. Data in (**C**) and (**D**) is normalized to body weight (BW). Data are mean (SD), n = 10/group (corresponding to n = 3-5 females and n = 5-7 males per group) *: $p < 0.05$, and ***: $p < 0.001$, two independent experiments. KO - knock-out.

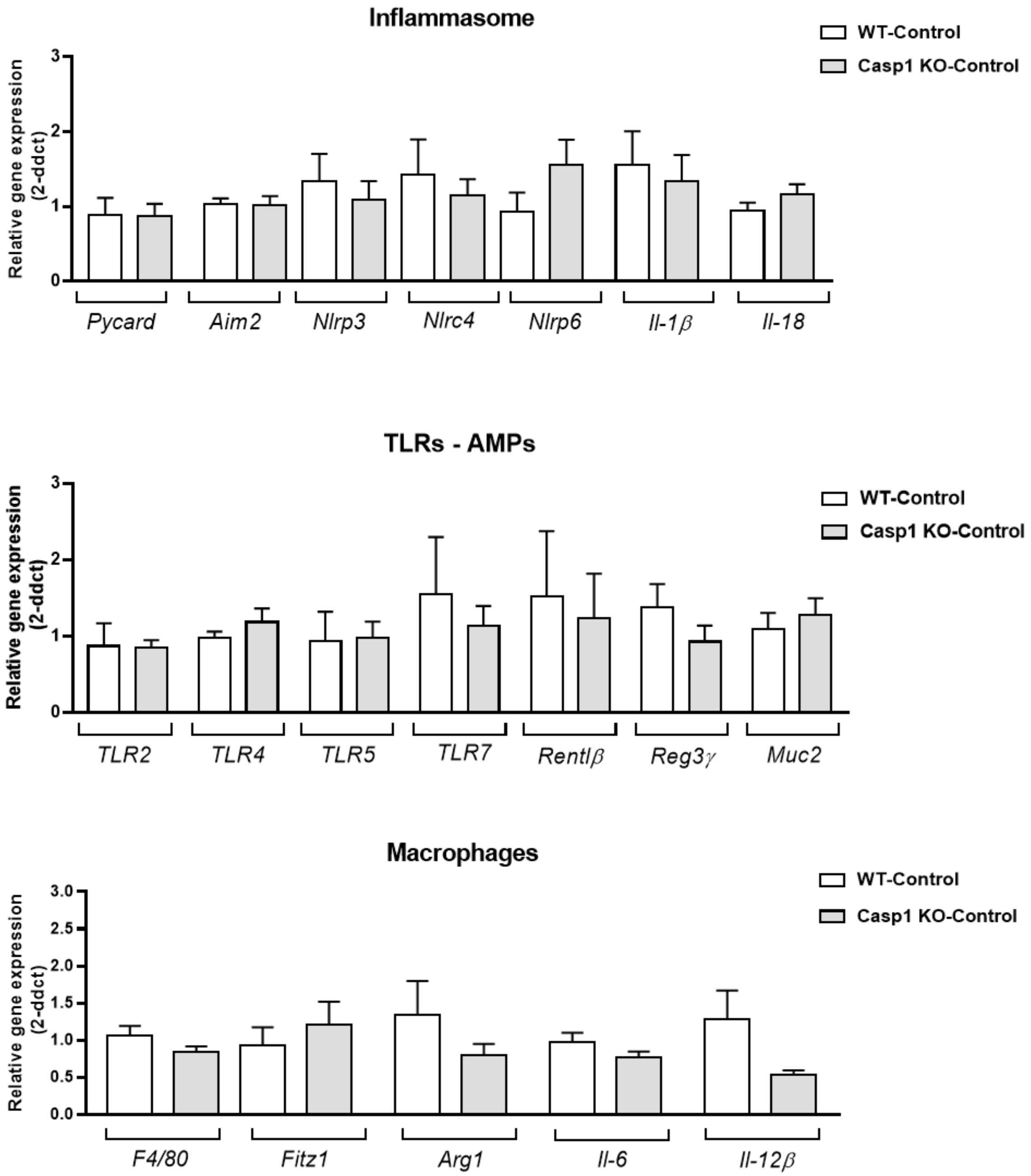

Supplementary Figure S2. Representative RT-qPCR expression of genes associated with Inflammasome (*Pycard, Aim2, Nlrp3, Nlrc4, Nlrp6, Il-1β and Il-18*); Toll like receptors (*TLR 2, -4, -5 and -7*); antimicrobial peptides AMPs (*Rentlβ and Reg3γ*), the mucus layer component *Muc2* and macrophage M1/M2 signature (*F4/80, Fitz1, Arg1, Il-6 and Il-12β)* in the colon of wild type (WT) and Casp1 KO control groups. Data are mean (SEM). *n* = 4-10/group.

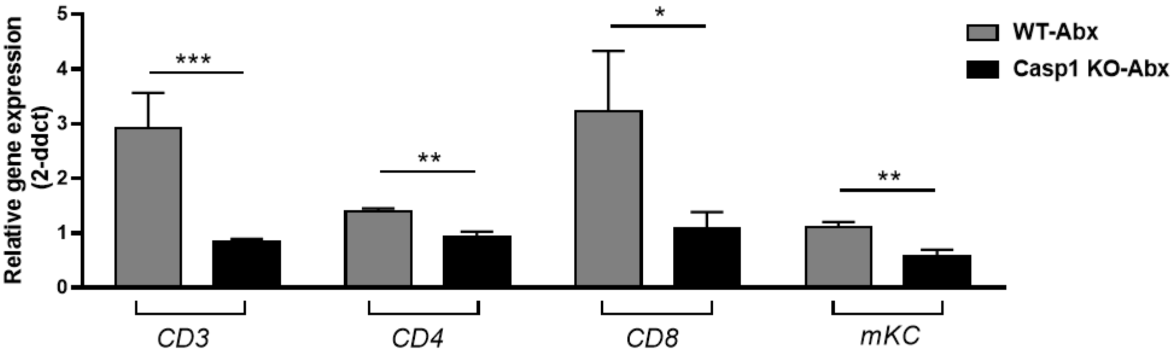

Supplementary Figure S3. Representative RT-qPCR expression of the T cells markers CD3, CD4 and CD8 and the neutrophil chemoattactant mKC of wild type (WT) and Casp1 KO antibiotic (Abx) treated groups. Data are mean (SEM). *n* = 7-9/group. *: $p < 0.05$; **: $p < 0.001$; ***: $p < 0.001$.

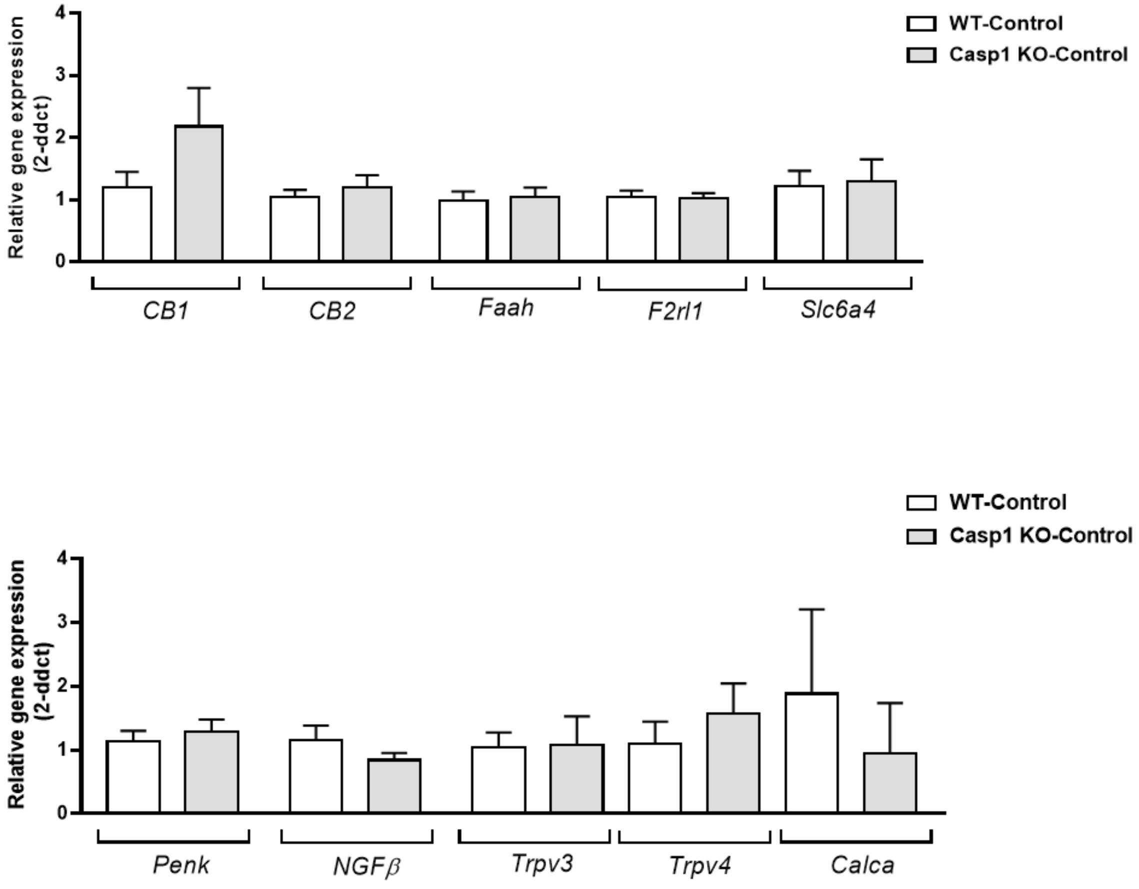

Supplementary Figure S4. Representative RT-qPCR expression of colon nociceptive markers; the endocannabinoid system (*CB1*, *CB2* and *Faah*), the protease-activated receptor 2 (PAR2, *Fr2l1*), serotonin transporter (*Scl6a4*), the opiod peptide pro-enkephalin (*Penk*), the neurothrophin (*NGFβ*), the vanilloid system (transient receptor potential, *Trpv3* and *Trpv4),* and calcitonin related polypeptide alpha (*Calca*) in the wild type (WT) and Casp1 KO control groups. Data are mean (SEM). *n* = 3-9/group.
